# Effectiveness of Activated Charcoal Toothpaste vs. 6% Hydrogen Peroxide Whitening Pen—An In Vitro Study

**DOI:** 10.3390/dj13050216

**Published:** 2025-05-19

**Authors:** Elena Bardellini, Silvia Marchetti, Alessandra Bordanzi, Simone Zanini, Alessandra Majorana, Giulio Conti

**Affiliations:** 1 Department of Medical and Surgical Specialties, Radiological Sciences and Public Health, School of Dentistry, University of Brescia, 25123 Brescia, Italy; s.marchetti009@unibs.it (S.M.); a.bordanzi@unibs.it (A.B.); s.zanini004@studenti.unibs.it (S.Z.); alessandra.majorana@unibs.it (A.M.); 2 Head and Neck Department, Department of Surgery, Dentistry, Pediatrics and Gynecology, University of Verona, 37129 Verona, Italy; giulio.conti@univr.it

**Keywords:** tooth whitening, hydrogen peroxide, activated charcoal toothpaste

## Abstract

**Background:** Tooth whitening is a widely sought-after cosmetic procedure, with various at-home and professional treatments available. This study compares the whitening efficacy of an activated charcoal toothpaste and a 6% hydrogen peroxide whitening pen under controlled in vitro conditions. **Methods:** Twenty freshly extracted human teeth were stained with a coffee solution and divided into two groups. Group A underwent daily applications of activated charcoal toothpaste for 30 days, while Group B received a single 5 min application of a 6% hydrogen peroxide whitening pen. Tooth color was assessed using the VITA Classical A1-D4 Shade Guide at baseline, mid-treatment, and post-treatment for Group A and at baseline and immediately after treatment for Group B. **Results:** The activated charcoal toothpaste exhibited a gradual whitening effect, with the most significant improvements occurring within the first two weeks (*p* < 0.01), after which the whitening effect plateaued. In contrast, the hydrogen peroxide whitening pen produced immediate and substantial whitening (*p* < 0.001). Statistical analysis using the Wilcoxon signed-rank test and Mann–Whitney U test confirmed the superior efficacy of the hydrogen peroxide treatment. **Conclusions:** The hydrogen peroxide whitening pen was significantly more effective in achieving rapid and substantial whitening compared to the activated charcoal toothpaste, which provided gradual but limited improvements. Further clinical studies are needed to evaluate the long-term color stability.

## 1. Introduction

Tooth whitening, also known as dental bleaching, is a widely practiced aesthetic procedure aimed at lightening the color of discolored or stained teeth. It is appreciated for being non-invasive, effective, relatively low-cost, and well-tolerated by patients [1]. Whitening systems can be categorized based on the type of chemical agent used, its concentration, method of application, and frequency of use [2]. Treatments may be performed in a professional setting or at home using over-the-counter (OTC) products. In both contexts, the active agents—primarily hydrogen peroxide (HP) and carbamide peroxide (CP)—act by releasing reactive oxygen species that oxidize chromogenic compounds in enamel and dentin, thereby producing a whitening effect [2].

In-office treatments typically involve high-concentration peroxide gels (30–40%), sometimes activated by light or laser and can achieve dramatic results in a single session. In contrast, at-home products contain lower concentrations of peroxide and require prolonged or repeated applications to reach satisfactory results. While slower, they are safer for unsupervised use and more accessible for patients seeking convenience or cost-effectiveness [3,4].

Among OTC solutions, whitening toothpastes represent one of the most accessible and frequently used options. Their whitening efficacy, however, is generally limited to the removal of extrinsic stains through mechanical action. These formulations contain abrasive agents—such as hydrated silica, calcium carbonate, alumina, or sodium bicarbonate—that polish the enamel surface and help eliminate pigmented biofilm and food residues. The degree of abrasiveness is often quantified by the RDA (Relative Dentin Abrasivity) index, which is typically higher in whitening toothpastes [5,6]. While they are effective in improving surface appearance, they are not capable of altering intrinsic discoloration. In recent years, whitening toothpastes containing activated charcoal have gained popularity due to their perceived natural origin and high absorptive capacity. Activated charcoal is a fine black powder obtained by carbonizing organic materials (e.g., coconut shells or wood) at high temperatures, resulting in a highly porous structure [7,8,9]. Its mode of action is thought to rely on the adsorption of surface pigments and mechanical removal during brushing. However, the scientific evidence supporting its efficacy remains limited and contradictory. Some studies suggest minor improvements in tooth color, while others raise concerns about its abrasive potential and lack of effect on intrinsic stains [7,8,9].

In addition to toothpastes, mouthwashes containing whitening agents have been introduced, including those formulated with activated charcoal. These rinses claim to offer adjunctive benefits by reaching interproximal areas and enhancing overall oral hygiene. However, the current literature suggests that charcoal-containing mouthwashes provide negligible or no added whitening benefit when used in combination with whitening toothpastes [10]. Moreover, concerns have been raised regarding their safety, potential to reduce enamel bond strength, and lack of proven efficacy [11,12]. As such, their role remains controversial and unsupported by robust clinical data.

Among newer OTC modalities, whitening pens have emerged as a convenient, portable alternative for at-home bleaching. These devices usually contain moderate concentrations of HP (typically 6–10%) in gel form, which is applied directly to the tooth surface via a brush-tip applicator [13]. Unlike toothpastes, pens function through direct chemical oxidation and can reach intrinsic discolorations. These pens offer the precise application of a thin layer of gel that adheres closely to the enamel through a brush applicator, potentially reducing HP permeability [13].

Their appeal lies in their simplicity of use, rapid application times (as short as five minutes), and targeted treatment capability. Whitening pens stand out for their convenience and ease of use, making them a popular choice among consumers. However, their effectiveness and safety are influenced by their formulation. Variability in their compositions—such as HP concentration and pH levels—significantly impacts their safety profiles. Despite their increasing popularity, studies evaluating their comparative effectiveness and potential drawbacks—such as uneven distribution, gingival irritation, or transient enamel dehydration—remain limited [14].

The long-term effectiveness of any whitening method may also be influenced by various factors, including dehydration-induced brightness, dietary habits, and the natural tendency of teeth to regain chromogenic molecules over time. For these reasons, many patients seek maintenance strategies involving the regular use of whitening toothpastes or periodic touch-ups with peroxide-based products [1,2,3,5].

The aim of this in vitro study was to compare the whitening efficacy of two popular at-home OTC products: an activated charcoal-containing toothpaste and a 6% hydrogen peroxide whitening pen. Extracted human teeth were artificially stained and treated under controlled laboratory conditions to evaluate and quantify changes in shade and brightness over time. This experimental model was designed to assess the absolute whitening effect—defined as the ability to produce a measurable change in tooth shade and brightness—in the absence of saliva and other intraoral variables.

## 2. Materials and Methods

Twenty recently extracted permanent teeth were collected from patients attending the Dental Clinic in Brescia, Italy. All teeth were thoroughly cleaned of any soft tissue and preserved in a 0.1% thymol solution, prepared using pharmaceutical-grade thymol powder (Sigma-Aldrich, Munich, Germany) and stored at 4 °C for a maximum of two months prior to use.

### 2.1. Sample Preparation

Extracted permanent teeth were arranged on plaster models to simulate the upper (Group A) and lower (Group B) dental arches. To induce staining, all specimens were immersed for 60 h in a coffee solution prepared by dissolving 1.8 g of coffee in 150 mL of boiling water. The solution was allowed to cool to room temperature before immersing the teeth.

### 2.2. Whitening Procedure

Group A underwent a whitening protocol using an activated charcoal toothpaste (Black is White^®^ Toothpaste, Curaprox^®^, Kriens, Switzerland), with an RDA of approximately 50. The toothpaste was applied with a medium-bristle toothbrush under controlled conditions. A calibrated force gauge was used to ensure a standardized pressure of 200 g was maintained throughout the brushing process. Each session lasted for 5 min and was per-formed once daily over a period of 30 days.

Group B received a single application of a whitening pen (QuickWhite^TM^ Pen, QuickWhite Ltd., Canterbury, UK) containing 6% hydrogen peroxide. The whitening agent was applied for 5 min following the manufacturer’s instructions.

For both groups, the whitening treatment was limited to the buccal surfaces of the teeth, in accordance with standard clinical protocols that typically exclude palatal and lingual surfaces.

### 2.3. Color Evaluation

The whitening efficacy was evaluated directly on the teeth using the VITA Classical A1-D4 Shade Guide. In Group A (charcoal toothpaste), shade assessments were performed at three time points: baseline (before treatment), mid-treatment (after 7 days), and post-treatment (after 30 days). In Group B (hydrogen peroxide whitening pen), shade assessments were conducted at two time points: baseline and immediately after the whitening agent application. Shade assessments were performed under standardized daylight simulation conditions (D65 light source) at a fixed time each day to minimize variability. All color determinations were performed by a single trained examiner (S.Z.) to ensure consistency. To document the process and provide a visual record, photographs of the specimens were taken at each assessment point using a high-resolution digital camera (Canon EOS 5D Mark IV, Canon Italia S.p.A., Milan, Italy) equipped with a macro lens (Canon EF 100 mm f/2.8 L Macro IS USM, Canon Italia S.p.A., Milan, Italy) and ring flash (Canon MR-14EX II Macro Ring Lite, Canon Italia S.p.A., Milan, Italy) to ensure consistent lighting and high-quality images.

### 2.4. Statistical Analysis

The primary outcome measure was the change in tooth shade before and after whitening, assessed using the VITA Classical A1-D4 Shade Guide. Shade scores were recorded individually for each tooth rather than as a single averaged value per group. Given the ordinal nature of the shade scale, data were analyzed using non-parametric statistical tests. The Wilcoxon signed-rank test was applied to compare pre- and post-treatment shade changes for each tooth within the same group. The Mann–Whitney U test was used to compare whitening effectiveness between the two groups on a per-tooth basis. The significance level for all statistical analyses was set at α = 0.05. Data were analyzed using STATA17 (StataCorp LLC, College Station, TX, USA).

## 3. Results

### 3.1. Group A: Charcoal Toothpaste

The initial and final shades of the upper arch teeth (treated with charcoal toothpaste) are summarized in Table 1.

**Figure 1:**
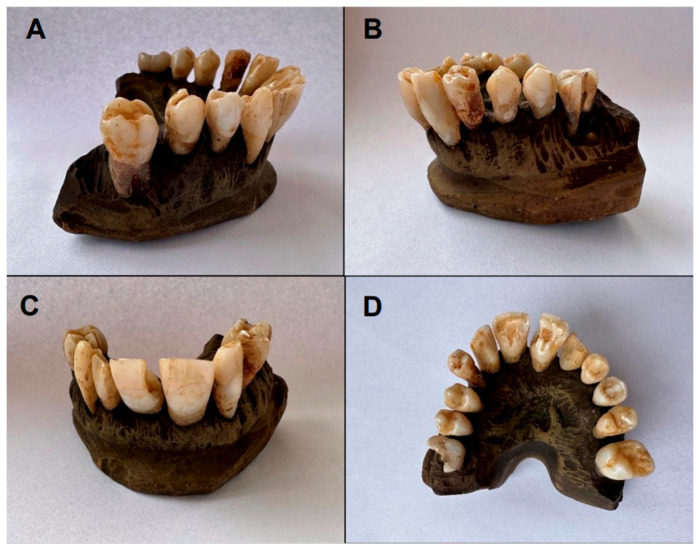
Initial color of upper arch teeth (Group A): (**A**,**B**) lateral view; (**C**) frontal view; (**D**) occlusal view.

**Figure 2:**
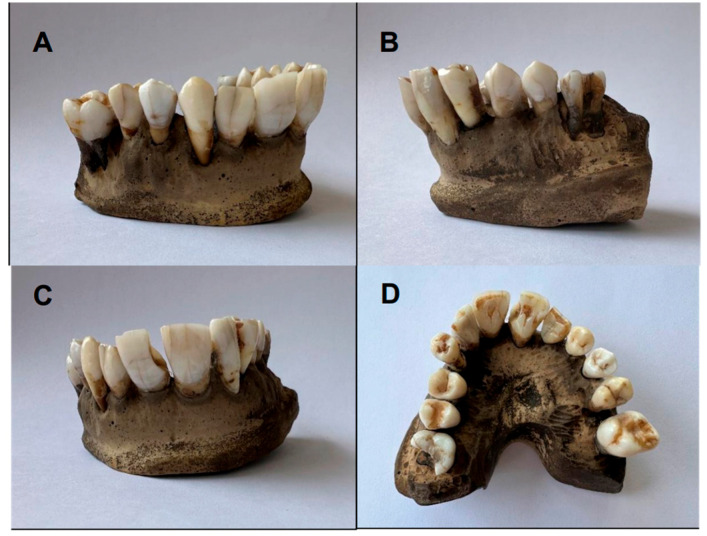
Final color of upper arch teeth (Group A): (**A**,**B**) lateral view; (**C**) frontal view; (**D**) occlusal view.

The initial and final colors of the upper arch teeth (after 30 days of bleaching with charcoal toothpaste) are shown in Figure 1 and Figure 2, respectively.

### 3.2. Group B: 6% Hydrogen Peroxide Whitening Pen

The initial and final shades of the lower arch teeth (treated with 6% HP whitening pen) are summarized in Table 2.

**Figure 3:**
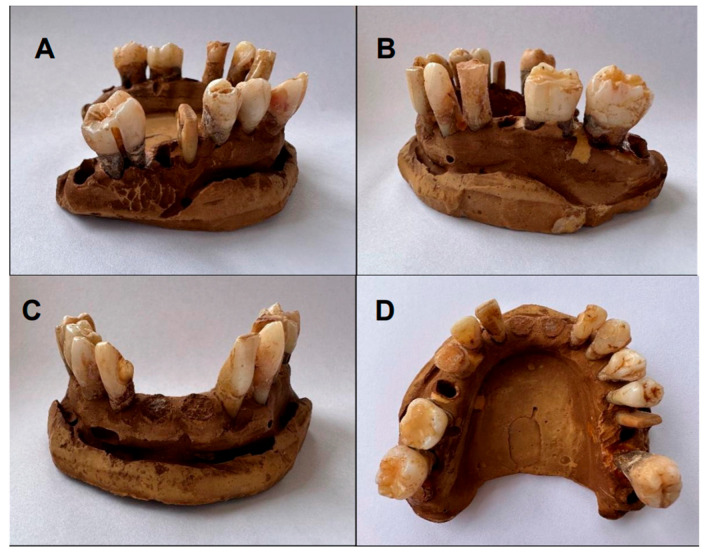
Initial color of lower arch teeth (Group B): (**A**,**B**) lateral view; (**C**) frontal view; (**D**) occlusal view.

**Figure 4:**
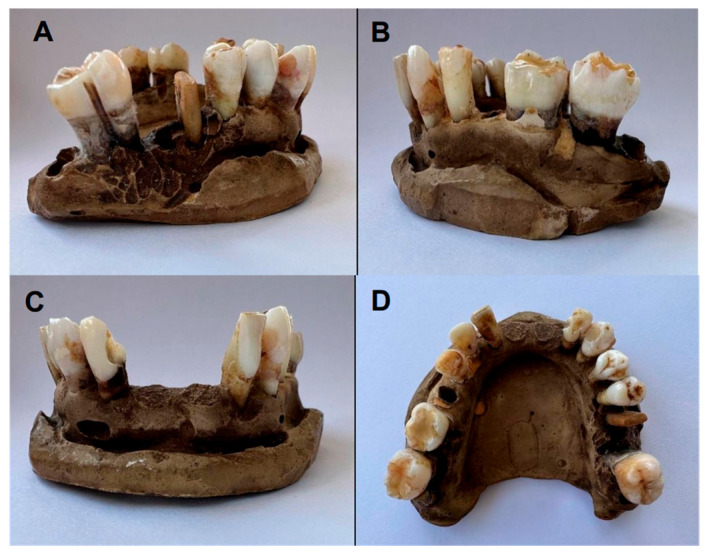
Final color of lower arch teeth (Group B): (**A**,**B**) lateral view; (**C**) frontal view; (**D**) occlusal view.

The initial and final color of the lower arch teeth (after a single application of the 6% HP whitening pen) is shown in Figure 3 and Figure 4, respectively.

A Wilcoxon signed-rank test was performed to compare the initial and final shade values within each group, revealing a statistically significant improvement in tooth color over 30 days for the charcoal toothpaste group (*p* < 0.01) and immediately after treatment for the hydrogen peroxide whitening pen group (*p* < 0.01). A Friedman test was conducted to evaluate shade progression at multiple time points (Day 7, 14, 21, and 30) within the charcoal toothpaste group, confirming a significant overall whitening effect (*p* < 0.001). Additionally, a Mann–Whitney U test was used to compare the whitening efficacy between the two groups, demonstrating the superior whitening potential of the hydrogen peroxide treatment in achieving both noticeable color change and enhanced brightness (*p* < 0.01).

## 4. Discussion

The findings of this study offer valuable insights into the comparative whitening efficacy of an activated charcoal toothpaste and a 6% HP whitening pen evaluated under controlled in vitro conditions. The two OTC products demonstrated significantly different whitening behaviors in terms of both onset and intensity of action. The results revealed significant differences in the whitening performance of these two over-the-counter treatments, particularly in terms of the onset of action and overall effectiveness.

The following is a detailed account of the weekly changes observed in the group treated with the activated charcoal toothpaste. After 7 days of application, a perceptible improvement in tooth shade was noted in the majority of samples, indicating early whitening activity. An exception was observed in the upper left first molar (tooth 2.6), which retained visible dark staining despite treatment. This limited response may be associated with the tooth’s prior endodontic treatment, potentially affecting its susceptibility to whitening. By Day 14, a generalized enhancement in tooth shade was evident (*p* < 0.01). However, the upper left first premolar (tooth 2.4) and the upper left lateral incisor (tooth 2.2) had already reached their final shade by Day 7, with only minimal further brightness observed, possibly due to the continued action of the charcoal. Notably, tooth 2.6—initially non-responsive—showed a marked improvement from shade C3 to C2 along with increased brightness compared to the first week. On Day 21, color changes appeared to stabilize, with the most pronounced improvements occurring within the first two weeks. During the third week, tooth shades remained largely consistent, with only slight brightness gains in a few cases. By the end of the treatment (Day 30), shades were generally unchanged from Day 21, indicating that the whitening effect had plateaued. Tooth 2.6 represented the only exception, transitioning from C2 to C1 with a notable increase in brightness. However, minor residual stains remained in the cervical and middle thirds of the crown.

Overall, the activated charcoal toothpaste exhibited a gradual whitening effect over the 30-day period, with the most substantial changes occurring within the first two weeks. This supports the hypothesis that the mechanical and absorptive properties of charcoal contribute to the removal of extrinsic stains, primarily through surface pigment adsorption and elimination during brushing. Nevertheless, the plateau observed after two weeks suggests limited capacity to address intrinsic discoloration. The persistence of stains—especially in teeth with prior endodontic procedures—reinforces the notion that charcoal-based whitening primarily affects surface-level pigmentation without penetrating the deeper enamel or dentin layers.

Evidence on the efficacy of charcoal-based whitening toothpastes remains limited. Dionysopoulos et al. [10] emphasized the scarcity of high-quality data in this area. In a comparative study, Vaz et al. [15] reported that a charcoal-containing toothpaste was more effective than a regular toothpaste (control group) but less effective than formulations incorporating microbeads, HP, or blue covarine. In contrast, Palandi et al. [16] found that an activated charcoal-based product failed to improve tooth color and might even alter enamel surface characteristics. Similarly, Brooks et al. [17], in a recent literature review, concluded that there is insufficient evidence to support the safety and whitening efficacy of charcoal-based toothpastes.

Activated charcoal, a nanocrystalline form of carbon with a high surface area and nanometer-range porosity, is widely used as an adsorbent in various fields [10,15]. Its application in oral care relies on its presumed ability to trap surface stains within its porous structure, which are then removed through brushing. However, scientific support for this mechanism remains inadequate. It has been proposed that the whitening effect of charcoal is primarily due to its abrasiveness, similar to conventional toothpastes, rather than any intrinsic chemical action [18]. Accordingly, some authors suggest its use only for maintaining post-bleaching tooth shade by delaying the reappearance of surface stains [10,19]. Other factors, such as toothbrush type, brushing technique, and duration, may exert a greater influence on cleaning and whitening outcomes than the toothpaste composition itself [10,20]. In the present study, these variables were standardized during all brushing procedures.

The abrasive potential of charcoal-containing toothpastes is believed to depend on the source, processing method, and particle size distribution of the charcoal used [10,20]. For whitening toothpastes to be both effective and safe, their abrasive content should facilitate cleaning while preserving enamel integrity [10,21]. Several studies have reported relatively high abrasiveness in charcoal-based oral hygiene products [10]. Dionysopoulos et al. [10] demonstrated that charcoal-containing toothpaste produced a more pronounced whitening effect than regular formulations. However, brushing with this product also caused significant alterations in enamel surface morphology, including the formation of deep craters and increased roughness. These effects contrast with those induced by standard toothpastes containing hydrated silica, highlighting the impact of differing abrasive agents.

The efficacy and safety of activated charcoal toothpastes remain controversial. Some studies suggest limited whitening potential and emphasize risks such as enamel abrasion [22]. Whitening toothpastes typically achieve their effect through enhanced surface cleaning, driven by either abrasive components or chemical additives. A recent systematic review and meta-analysis by Jamwal et al. [23] found that while these products may lighten tooth shade by one to two units, they also tend to increase enamel roughness and decrease microhardness. The authors emphasized the need for further research to identify whitening agents that are both effective and safe for long-term use. Supporting this, Tomas et al. [24] reported that charcoal-based toothpastes offer inferior whitening outcomes compared to alternative agents and pose greater risks due to their high abrasiveness. Koc et al. [25] further stressed that the abrasive effects of such formulations should not be overlooked.

In contrast, the application of the 6% HP whitening pen resulted in immediate and substantial shade improvements (Table 2) (*p* < 0.01). Additionally, the treatment induced a marked increase in brightness and led to the complete removal of both superficial and deeper stains. This rapid whitening effect is attributable to the oxidative mechanism of HP, which produces free radicals capable of disrupting the conjugated double bonds of pigmented molecules. The complete removal of discolorations confirms the superior penetrative capacity of HP, which is able to reach both the enamel and dentin layers and induce chromatic changes at the molecular level. These findings are consistent with prior studies that have demonstrated the high efficacy of HP-based whitening agents, even at moderate concentrations and with short application times [26,27,28]. A closer examination of peroxide-based whitening products suggests that their rapid action may partly stem from the chemical instability of HP, which tends to release all its active components within a short timeframe—typically 30 to 60 min. While this results in immediate whitening effects, it may limit sustained interaction with the organic matrix of dental tissues, potentially affecting the longevity of the outcome. In contrast, CP—a peroxide derivative not evaluated in this study but widely used in clinical practice—decomposes more gradually. Approximately 50% of its content is converted into HP within the first 2 to 4 h, followed by a slower release over subsequent hours. This prolonged release allows for continuous oxidative action, thereby potentially enhancing the breakdown of intrinsic stains within the dentin [29,30,31]. Consequently, while HP demonstrated superior short-term outcomes in this study, CP-based agents may also offer advantages in terms of long-term color stability [29,32].

Another important observation in this study concerns the impact of treatment duration on whitening outcomes. While the activated charcoal toothpaste required extended use to produce visible effects, the HP pen delivered substantial whitening after a single five-minute application. This distinction is especially relevant from the standpoint of patient compliance, as users may be more inclined to adhere to shorter, more efficient protocols. Furthermore, the significant increase in brightness observed in the HP group may also reflect a transient dehydration effect—commonly reported during the early stages of bleaching procedures.

These findings emphasize the importance of tailoring whitening strategies to individual patient needs and expectations. Activated charcoal toothpaste may be appropriate for individuals seeking gradual surface stain removal with minimal intervention, whereas HP-based treatments represent a more suitable option for those desiring rapid and dramatic improvements. However, potential adverse effects—such as tooth sensitivity and enamel demineralization—should be considered when recommending peroxide-based products, particularly with repeated or prolonged use.

Finally, it is important to acknowledge the limitations of this study’s in vitro design. The absence of saliva, dietary influences, and natural remineralization processes may restrict the clinical applicability of the results. Future in vivo studies are necessary to evaluate long-term color stability, the interaction of whitening agents with the oral environment, and patient-centered outcomes such as satisfaction, compliance, and potential side effects. Additionally, the use of spectrophotometric tools and comparisons across different formulations and application protocols will be essential to optimize both efficacy and safety in at-home whitening treatments.

## 5. Conclusions

This in vitro study provides a comprehensive comparison between an activated charcoal toothpaste and a 6% HP whitening pen, highlighting key differences in their mechanisms of action, onset of effect, and overall whitening performance. The activated charcoal toothpaste demonstrated a gradual and surface-limited whitening effect, primarily through mechanical stain removal, with most changes occurring within the first two weeks of use. In contrast, the HP pen induced rapid and more pronounced whitening, including the removal of intrinsic stains, owing to its deeper oxidative action on dental tissues.

While charcoal-based products may be suitable for individuals seeking mild, progressive whitening with minimal intervention, peroxide-based treatments offer superior results in a shorter timeframe. However, their use should be guided by clinical supervision, especially in repeated applications, due to potential concerns reported in the literature.

## Figures and Tables

**Table 1:** Initial and post-treatment shade changes following the application of activated charcoal toothpaste in Group A (upper arch).

Group A (Upper Arch)					
Tooth Number	Initial Color (VITA Classical A1-D4 Shade Guide)	Day 7	Day 14	Day 21	Day 30
2.6	C3	C3	C2	C2	C1
2.4	C1	B1	B1	B1	B1
2.3	B3	B2	B2	B2	B2
2.2	C3	C1	C1	C1	C1
2.1	C2	C1	A1	A1	A1
1.1	C2	C1	C1	C1	C1
1.2	C3	C1	A1	A1	A1
1.3	C4	B2	A1	A1	A1
1.4	C2	C1	A1	A1	A1
1.5	C1	A1	A1	A1	A1

**Table 2:** Initial and post-treatment shade changes following the application of 6% HP whitening pen in Group B (lower arch).

Group B (Lower Arch)		
Tooth Number	Initial Color (VITA Classical A1-D4 Shade Guide)	Final Color After Single Application
3.7	C3	C1
3.6	C3	B2
3.4	C4	A3
3.3	D2	B1
3.2	D3	A1
4.2	A4	A1
4.3	C3	incisal third B1 middle and cervical third C1
4.4	C1	B1
4.5	C4	C1
4.7	C3	C1

## Data Availability

The data that support the findings of this study are available from the corresponding author upon reasonable request.
